# Becoming Job-Ready? Narratives of Local Welfare-to-Work Programs and Client Experiences Across Differing Economic Contexts in California

**DOI:** 10.1177/10443894211048451

**Published:** 2021-12-11

**Authors:** Lucia M. Lanfranconi, Aditi Das, Joy Subaran, Patricia Malagon

**Affiliations:** 1 PhD, professor, Lucerne University of Applied Sciences and Arts, Switzerland; 2 PhD, consultant, Resource Development Associates, Oakland, CA, USA; 3 BA, research apprentice, University of California, Berkeley, USA; 4 BA, research associate, University of California, Berkeley, USA

**Keywords:** job-readiness, client experiences, organizational narratives, regional economic context, welfare-to-work, place-based inequities

## Abstract

Previous research on welfare-to-work exits has focused on individual client characteristics rather than local economic contexts. Drawing on a qualitative comparative case study design, this study enhances our understanding on how welfare-to-work organizational narratives and client experiences of becoming job-ready are shaped across two different economic contexts. In the disadvantaged economic context, a punitive welfare-to-work narrative is operational resulting in clients accepting precarious work. In the more privileged economic context, the individual responsibility narrative dominates as clients struggle to make ends meet. Our findings highlight how regional economic factors shape organizational narratives and impel clients to accept precarious low wage working conditions and unstable housing. Thus, there is a need for alternatives to welfare-to-work, such as unconditional, Universal Basic Income.

Welfare-to-work (WTW) has long been an issue in American social policy. Research suggests a correlation between economic conditions and WTW exits: Living in a more disadvantaged economic context will make welfare exits especially challenging (Grogger & Karoly, 2005). Local labor markets differ in economic trends and sociocultural historic contexts (Achdut & Stier, 2016). Temporary Assistance to Needy Families (TANF) recipients often transition into jobs that provide relatively low wages and unstable conditions (Loprest & Nichols, 2016). In a recent study, Chang et al. (2020) found varying service orientations across counties in California to be contingent on local labor market conditions. Counties with higher socioeconomic characteristics adopted an employment/training-oriented WTW approach compared with a more sanction-oriented system in disadvantaged counties. Besides, previous literature on WTW employment primarily focuses on individual client demographic characteristics (Achdut & Stier, 2016; Acs & Loprest, 2016; Hill, 2012). Research has called for studying the variance of place-based experiences, as it is important to acknowledge the spatial distribution of economic disadvantage, employment opportunities, and welfare utilization (Irving, 2008).

The current study draws on a qualitative comparative case study design to enhance our understanding of how the local economic context shapes WTW organizational narratives and client experiences of becoming job-ready. Organizational narratives refer to the spoken or written stories that operate within organizations, which refers to how an organization shapes client prospects of becoming job-ready (Clair, 2009; Short & Payne, 2020). The study builds on the theoretical framing of how policy designs are socially constructed and thereby influenced by prevailing ideas (Schneider & Ingram, 1993, 1997) such as the work-first organizational narrative or understandings of the client’s individual responsibility for charting their own self-sufficiency pathways. Social construction and policy design theory further presume that, based on such narratives, policy designs reinforce and perpetuate the socially constructed reality (Chang, 2019; Schneider & Ingram, 1993, 1997; Starke, 2020). Thus, local organizations, through organizational narratives, may be reproducing macro-level policy discourses, which in turn can shape the possibilities and experiences of WTW clients becoming job-ready. Using California as the study site, the study compares two local contexts, an agrarian context named Central-County (situated in the Central Valley) and an urban, technology-focused context named Bay-County (situated in the Bay Area) to shed light on the role regional variation plays in shaping job prospects for WTW organizational narratives and client’s experiences.

## Review of Related Literature on Economic Context, WTW Clients, and WTW Agencies

Previous research suggests that structural factors such as a shortage of living wage jobs, poor public transportation, or expensive childcare pose as major barriers to WTW clients (Danziger et al., 2013; Monroe & Tiller, 2001). Qualitative studies illuminate low-income single mothers in WTW struggling to receive welfare benefits, being sanctioned instead of being supported by TANF agencies (Seefeldt, 2017) and often earning wages below the poverty level without the opportunity to build on human capital (Cleaveland, 2007; Danziger et al., 2013).

Compared with other Organisation for Economic Co-Operation and Development (OECD, 2020) nations, the United States is infamously known for its poor employment protections, especially for low-wage labor. Employers further showcase strong hiring preferences for candidates who are more educated and have soft skills, prior work experience, and flexibility, with greater barriers for high school dropouts and minority recipients (Acs & Loprest, 2016). Studies have shown that state and local welfare agencies are continuing to explore ways to improve client engagement in work-related activities like structured job-search workshops, and participation incentives for TANF recipients, yet most agencies continue to operate on the work-first approach (Pavetti, 2018).

Irving (2008) conducted a comparative study on welfare exits in nonmetros (more disadvantaged) and other metros (less disadvantaged) and found important differences in TANF exiting behavior concluding that states should not adopt a “one-size fits all approach” to lower welfare caseloads. In another study, local labor market conditions and their change over time play an important role in explaining long-term employment and earnings patterns among single mothers (Achdut & Stier, 2016). While there has been little increase in the quality of jobs in heavily low-wage firms (Andersson et al., 2016), there has been a significant mismatch between the location of low-wage workers and available employment. These factors suggest that those who transfer from welfare to work will have difficulty moving out of low-wage work, thereby emphasizing the role of economic context and regional variation in shaping both local agency contexts and individual client experiences of becoming job-ready. The current study adds to this literature.

## California’s Labor Market and TANF Program

Before the Covid-19 pandemic, California’s job growth had been strong over the past decade. Even though income improved, the state’s economic polarization is on the rise and regional disparities exist. For instance, unemployment rates in inland and far Northern California are higher compared with the urban, coastal areas (Bohn et al., 2019). California’s TANF Program, titled California Work Opportunity and Responsibility for Kids (CalWORKs), assists some of the state’s low-income families and is one of the most inclusive, client-centered TANF programs across the nation (Stanczyk et al., 2018). CalWORKs requires adults to participate in a minimum number of hours per week between 20 and 35 depending on the family composition and age of children (California Department of Social Services [CDSS], 2019). CalWORKs has a robust subsidized employment program and operates Job Clubs to move clients from cash-aid to employment and also provides support for CalWORKs recipients enrolled in academic programs (Anderson et al., 2019). CalWORKs operates on a highly devolved governance structure, as it is administered by county welfare departments, under the supervision of the CDSS.

## Case Study

This study uses data that were collected as a part of a broader mixed-methods study on “Social Equity Within California’s WTW Program” (see Chang et al., 2020; Lanfranconi & Basaran, forthcoming; Lanfranconi et al., 2020a, 2020b). The study adopts a qualitative comparative case study design by juxtaposing two “most different” cases (Flyvbjerg, 2006). The two case-counties were selected through a process that entailed three key steps. First, *criteria selection*: After a thorough literature review, interviews were conducted with experts in academia and key CalWORKs state officials to determine the criteria for choosing two different counties (see Lanfranconi et al., 2020b). The key program and sociodemographic criteria selected, based on this information, were *WTW exemption*, *WTW sanction*, *non-White population*, *poverty rate*, and *political ideology.* Second, *county cluster selection*: A cluster analysis across 58 Californian counties was conducted (see Table 1; Lanfranconi et al., 2020b). Third, *case county selection*: Based on expert interviews conducted, three counties were chosen per cluster and contact was established with each county’s WTW Director to determine their interest in participating in the study. Finally, two county directors agreed to be a part of the study.

**Table 1.** Characteristics of Bay-County and Central-County, Adjusted.

County characteristic	Bay-County	Central-County
Exemption	26%	22%
Sanction	15%	34%
Non-White	67%	71%
Democrat	73%	51%
Unemployment	3%	10%
Poverty	9%	23%
Urbanization	412	132
Income (US$)	116,200	50,100
Rent (US$)	2,100	1,000
CalWORKs benefit (US$)	1,191	1,122
WTW Service orientation	Training-orientation	Sanction-orientation
Job examples from WTW flyers	Tesla, Amazon, Philz, order picker, administrative assistant, caregiver	Sales clerk, chicken catcher, childcare assistant, receptionist, retail associate

*Note.* WTW = welfare-to-work; CDSS = California Department of Social Services; CalWORKs = California Work Opportunity and Responsibility for Kids.

1. WTW exemption/sanction as a percentage of WTW population source: CDSS WTW25 Monthly Activity Report All (Other) Families, 2018.

2. Non-White rate source: The Census Reporter, Table B03002 (American Community Survey (ACS) 2017 5-year data).

3. Democrat source: Percentage of voters casted for the 2016 Democrat presidential candidate.

4. Urbanization source: Number of people per square mile (population density); The 2010 Census Population Density Data.

5. Unemployment rate source: Monthly Labor Force Data for Counties Annual Average 2018; State of California, Employment Development Department (REPORT 400 C).

6. Poverty rate source: The Census Reporter, Table B17001 (ACS 2017 5-year data).

7. Median household income (in 2018 dollars), 2014–2018 source: U.S. Census Bureau, Quick Facts per County.

8. Median gross rent, 2014–2018 source: U.S. Census Bureau, Quick Facts per County.

9. CalWORKs benefit levels for a family of four in rural and urban areas source: CalWORKs data 2019.

10. County WTW service orientation, based on a cluster analysis of Chang et al. (2020).

11. Job examples from WTW flyers and employment opportunity resources, such as orientation videos, workshop materials, and county job board page.

Table 1 presents the characteristics of the two selected counties. To ensure anonymity, the case counties were named Bay-County and Central-County. Bay-County is characterized as being highly urban, diverse, and largely democratic in its political ideology within proximity to Silicon Valley. With a growing technology industry, Bay-County has a lower unemployment and poverty rate but has a higher standard of living compared with other parts of California. The county’s WTW service orientation has been categorized^1^ as being training-oriented, and this cluster stands out for possessing the highest training service utilization rate (Chang et al., 2020). In contrast, Central-County is characterized as largely rural with a more conservative ideology compared with Bay-County. It is further situated in an agricultural community that has a higher unemployment and poverty rate. The county’s WTW service orientation is more sanction-oriented, which means it is characterized by a relatively high sanction rate (Chang et al., 2020).

Bay-County has been characterized as having a more privileged economic context (low poverty and unemployment rate, high median household income, etc.), while Central-County has a more disadvantaged economic context (high poverty and unemployment rate, low median household income, etc.). However, as stated on the official CalWORKs website, benefit levels are not necessarily adjusted as per the local context:

they’ll give your family a slightly higher benefit if you live in an urban area (a city) than if you live in a rural area (the country). While a family of four in region 1 (urban regions) can receive up to $ 1,181, the same family in region 2 (rural regions) receives up to $ 1,122.^2^

## Data

The fieldwork was completed by the first author in Central-County in October 2019 and in Bay-County in December 2019. The first author spent 2 weeks in each of the county sites to complete the interviews, observations, and collection of WTW documents. The study received institutional review board (IRB) approval from both the author’s university and county sites. All observations, interviews, and material at the county sites were collected with the informed consent of the participants. Each agency develops their own WTW workshops and customized material such as client flyers and PowerPoint presentations.

To understand what narratives local WTW agencies operate on, the study triangulates (see Jick, 1979) four primary data sources across both counties to get a more complete picture and until the principle of saturation was reached: (a) 27 documents from WTW workshops, including PowerPoint presentations and flyers given to WTW clients; (b) 32 flyers and informational resources given to clients during the CalWORKs program intake and orientation; (c) observation notes from three WTW workshops (one in Central-County and two in Bay-County); and (d) interviews with three workshop trainers following the workshops. The workshops were held at the respective WTW agencies by a trainer employed by the agency with three to nine participants in attendance. Based on the earlier interviews with managers and directors from the respective agencies (see Lanfranconi et al., 2020b), workshops that were mandated for all clients were selected. With the aim of ensuring diversity in the data, an additional workshop was chosen at Bay-County as multiple workshops are offered at this site.

To understand clients’ diverse experiences of becoming job-ready, the sample comprised 14 in-person, semistructured interviews (seven per county) conducted with the heads of families enrolled in CalWORKs either at the local agency or at the client’s home. The interviewees were chosen based on a qualitative sampling plan, ensuring racial and ethnic diversity among clients, aiming to also capture diversity of opinions and experiences among study participants (Kelle & Kluge, 2010; Mason, 2010; TESOL International Association, 2021).^3^

The sample comprises a majority of female clients (*n* = 12 out of 14) in line with the average gender-balance in CalWORKs (wherein 10% of clients are male, CDSS, 2019). The clients hail from diverse family constellations that include single as well as married households and have between one and five children. All the participants were high school graduates or equivalent, and 57% of Central-County participants and 85% of Bay-County participants had additional specialized training or some college. Almost all clients had experienced significant crises such as domestic violence, mental health, homelessness, incarceration, or drug abuse.

At the time of data collection, these selected clients were enrolled in local TANF agencies across Bay-County and Central-County for at least 1 month and were in the process of becoming job-ready. They were receiving cash aid for their families and were either participating in a WTW workshop (also called job-readiness or job-search), or gaining work experience through subsidized and unsubsidized employment, or pursuing a higher educational degree. Clients were recruited during their visits to the welfare agencies and were provided details of the study, signed an informed consent, and received a US$25 gift card as an incentive to participate. The interviews were audio-recorded with participant consent, lasting between 40 minutes and 1½ hours, and later transcribed for analysis.

## Analytical Approach

Drawing on a combination of critical discourse analysis (CDA; Keller, 2011; Turgeon, 2018) and content analysis (Kelle & Kluge, 2010), the interviews, observations, and documents were analyzed to juxtapose emerging patterns from embedded narratives operating within local WTW organizations as well as client experiences. The analysis comprised two major steps.

First, utilizing MAXQDA, a computer-assisted qualitative data analysis software, all data sources were deductively coded into six main codes: (1) *Equity–Inequity*, (2) *Differences*, (3) *Problems/Challenges*, (4) *Responsible actors*, (5) *CalWORKs programming and experiences*, and (6) *Language (for documents)*. The analysis began with deductive coding as proposed by Kelle and Kluge (2010) and based on the overall research question on social (in)equity in WTW as well as the semistructured interview grid (see Chang et al., 2020; Lanfranconi et al., 2020b). All documents were further inductively coded to generate various subcodes. To ensure a common understanding and interpretation of each subcode, three different researchers involved in the coding-process, discussing their interpretations in regular team-meetings as well as using memos in the commonly used MAXQDA file of each subcode. For this article, we generated and analyzed the following codes: (2) *Differences*, with subcodes *race*, *ethnicity*, *migration*, *gender*, *age*, and so on (to compare both client experiences and organizational narratives), (3) *Problems/Challenges*, with subcodes *economic hardship*, *unemployment*, *housing*, and so on (mainly to compare the client experiences), (5) *CalWORKs programming and experiences*, with subcodes *WTW intake*, *plan*, *subsidized employment*, *WTW workshop*, *work experience*, *education/vocational training*, and so on (to compare both client experiences and organizational narratives) and (6) *Language*: *emotional*, *supportive versus cold*, *complex* (mainly to analyze organizational narratives).

Second, after coding the data, citations were exported out of MAXQDA into an excel format to perform a comparative analysis across both counties. Major patterns from each county site were arranged into similarities and differences situated in the context of organizational narratives and client experiences. Dominant organizational narratives and client experiences were derived from the pattern table analysis and typical citations noted.

## Findings

Findings are presented case by case, presenting the dominant WTW organizational narrative as well as clients’ experiences of becoming job-ready within each respective county context.

### Case of Central-County

#### Organizational Narratives

Across both counties, WTW organizations’ narratives, as observed from the WTW workshop documents, and participant observations of those workshops, were that being a successful worker implies accepting any job, no matter how precarious the working conditions are. This narrative of “any job is better than no job” was found to be more dominant in Central-County, where clients are actively persuaded to accept any job in this context abound by high unemployment. For example, at a job-readiness workshop, the trainer wrote on the whiteboard the various reasons clients are better off working rather than being dependent on the system. The trainer stated, “Being on welfare, being broke, helpless, and bored with too much free time even though you receive a steady source of cash-aid and can spend predictable time with family; self-sufficiency is always a better option no matter what.” The trainer’s rationale for becoming self-sufficient was for clients to aspire for a better lifestyle and future, to teach their children about the importance of hard work, and to improve one’s self-esteem. While the trainer acknowledged that work can often be stressful and demanding, clients were urged to think about the big picture of how any job is better than the alternative, that is, being dependent on aid and dealing with arduous paperwork. Central-County clients were constantly reminded that

a job is a job. You would never quit [. . .]. Every job is better than no job even if unpaid [. . .]. Be comfortable with being uncomfortable. [. . .] Take responsibility instead of blaming [. . .]. Don’t put the fault on anybody else, [driving the point that clients ought to remain grateful to their employers, however challenging the job].

Across both counties, clients are asked to evaluate their own life choices while securing employment. These choices are about not having more children, getting married, and investing in their education. While this narrative is more dominant in Bay-County, it plays out also in Central-County. For instance, family planning was encouraged at a job-readiness workshop in Central-County. A male client was discouraged from having another child as he was reminded by the trainer that having more children is expensive and finding a home to rent is difficult. In the interview after the workshop, the trainer explained how she talks about this topic regularly in the workshops and tries to discourage clients from having more children:

A lot of times we’ll have that conversation. The more children that we have the busier we’re going to be, the less money we have because, they (the children) are expensive. It gets more and more expensive. So, I usually ask my clients, “Have you worked out family planning? What are your ideas about family planning?”

Besides this individual responsibility narrative, Central-County compared with Bay-County is relatively more cognizant of structural barriers clients faced while searching for work. For example, gender support groups have been created to teach new parents how to take care of their children. In addition, clients with a criminal record had access to an eight-page worksheet that contained advice on how to address a criminal background in their job application and provided a roster of jobs that hire people with felonies.

#### Client’s Experiences

Across both counties, clients are struggling to make ends meet as often the part-time and time-consuming nature of the work makes it hard for clients to financially support their families. Moreover, clients in subsidized and/or unsubsidized employment often described their working conditions as precarious, not adhering to standard schedules, disrespectful, and discriminatory (e.g., based on race or gender). Economic hardship while being on CalWORKs is a reality. A female single-earner with children speaks about her unstable working hours and how low wage work makes it hard for her to support her family:

One day when I really, really had nothing, my PG&E [power] went out. My job didn’t give me enough hours . . . They have unstable hours—it goes up and down.

While clients from both counties experienced workplace precarity and discrimination, Central-County clients specifically spoke of their unsafe and abusive work conditions, where employers were seen to take advantage of their labor. For instance, in the observed WTW workshops in Central-County, a female client mentioned that she had a miscarriage while handling dangerous chemicals at her workplace, where her employer, instead of taking responsibility, shifted the blame onto the client stating that the client chose to work there. In another case, a Hmong single mother who was pregnant with her fourth child described her experiences of being discriminated against while working at a CalWORKs subsidized job site:

They try to give you bad treatment to get rid of you. Because I just didn’t quit, they finally told me I wasn’t fit. It’s not a good thing because they only want profit, but some people are like that. They don’t want to give out too much money, they just want to make money. They even told me—“You’re invisible here. You don’t work here—you’re only here through the HSA program.” They didn’t really treat me like I was a worker either.

Across both county sites, clients discussed varying degrees of flexibility of the WTW program structure. Central-County clients describe how stressful it is to participate in 6-week, 4- to 6-hr mandatory workshops, and be sanctioned if they came in late or missed a workshop. One client recalled that, “There was a young lady, who was a single mom with a little girl and she didn’t have a babysitter for that day. [. . .] She had to start all over. She talked to me and she cried.” Apart from the WTW workshops, the work requirement pressures placed on clients were notable. For example, a white father of five and single-earner in Central-County describes his experience of balancing his required work hours, arduous paperwork, and spending time with his family:

There were times where I did drop out of the program because I would get a job and report, filling out the paperwork was a lot. It was too much having to work a job, worry about the stresses, and then come home and then write. It was too much. I had to keep up with my job, but worry about my job and family.

In Central-County, clients, compared with Bay-County clients, feel more “placed” in WTW activities. A single-earner stated, “They place you here, they place you there, they place you in a lot of places.” Other clients expressed being “held back” by the program. A Hispanic single mother of two said, “If I do have a job, I would stop all this [CalWORKs]. It’s kind of like, they kind of try to hold you back in a way.” This implies that CalWORKs was not providing the right stepping-stone for clients to advance their careers and improve their family’s well-being.

### Bay-County

#### Organizational Narratives

The work-first narrative is more present in Bay-County compared with Central-County, as there is an expectation for clients to adjust their schedules and behaviors as per their employer’s demand. For instance, in Bay-County, clients are told in the “How to Succeed” workshop materials to,

Be Flexible. When the company needs someone to change shifts, work weekends, put in some overtime, or work a different schedule, think about volunteering. Maybe it’s not your favorite job. However, it’s a paycheck . . . Stop complaining. Nobody likes complainers, regardless of how legitimate the complaints are.

During workshops, clients are often handed flyers that point to how clients ought to hone positive attitudes and self-disciplinary behaviors, including upkeeping a neat physical appearance while seeking employment: “Always communicate in a positive way and remember that your behavior talks—so smile!” thereby implying that clients ought to take control of their experience without externalizing blame to the local economic context in which they are located.

The individual responsibility narrative is more dominant in the Bay-County compared with the Central-County, where clients are encouraged to make better lifestyle choices for themselves such as not having more babies, getting married, or investing in one’s own education. Observations from a Bay-County WTW workshop called “Reality Check” highlight the steps that clients must take to make ends meet. For instance, clients completed an online activity that helps them think through their desired lifestyle and the kind of “financial choices” and life adjustments they need to make to aspire for that lifestyle. The online tool shows, for example, the cost of an additional child, which made participants resonate: “If they would show that in schools, maybe they (young people) would not have all these babies.” The workshop trainer explained after the workshop how important it is that clients have “realistic expectations” especially with regard to housing: “So they [the clients] have to change their expectations. I have a client right now she is stable, but she is living in her parents’ garage with four children, at least she is with her family.”

Another workshop trainer explained that sometimes he recommends clients move out of the county, due to the “high living-cost,” as he exclaimed, “We are only three miles away from the high-tech industry, but for our clients, the technology industry is so far away.” When asked about barriers in finding jobs, the trainer highlighted clients’ individual characteristics the most: “It’s hard for them to become financially stable. Mental health and criminal background are barriers.” The structural conditions that pose as barriers to becoming self-sufficient, such as a higher cost of living, were not factored into workshop content and material. For instance, in a WTW workshop called “Core 4” in Bay-County, all the workshop participants were mothers. However, the theme of balancing motherhood while finding a job was never discussed at the workshop.

#### Client Experiences

In Bay-County, the soaring rent prices have made it unaffordable to find a home, and many clients experience homelessness or discrimination as a renter. Other Bay-County clients expressed how nonstandard schedules and understaffing practices have detrimental effects on their physical and mental well-being. For example, a Hispanic single mother said,

It’s always busy and our staff isn’t fully staffed. It’s really hard when you don’t have everybody there, you’re working for three people when you’re supposed to work for one person. There were times when I didn’t have my lunch, I didn’t even have breaks. I was getting sick because of that.

In Bay-County, workplace bias based on race and/or gender was mentioned several times in clients’ experiences. For example, a single Black mother of two children and a victim of domestic violence was discriminated against by her employer:

The head of marketing gave me an impossible task because she knew I didn’t have the credentials for it . . . She’s probably thinking, “Who is this Black girl? She has no credentials for it.” . . . It was obvious that she didn’t want me there. It was racism.

The same client, together with many other Bay-County clients, talks about being homeless:

We lived in a house owned by the parents [of her husband who abused her]. As soon as I called the police, they put him in jail, the parents made me and the two children leave the house. As of October 14, we have been temporarily homeless.

In addition, clients have also been unfairly treated by landlords and agency workers.

The real problem in [name of the city in Bay-County] is that Black people can’t rent. . . There were some workers who didn’t put my information on because I wasn’t homeless enough. At some point, I was crying coming in here. They need to follow the rules and see my eligibility and not think about what I look like. I’m homeless, how homeless do you want me to be? I’m sleeping in my car, come follow me.

Bay-County grants more flexibility to clients compared with Central-County for adhering to WTW guidelines. Even though WTW workshops are mandatory for clients, the Bay-County workshop is shorter (1 week vs. 6 weeks), attendance is less strictly enforced, and clients can choose workshops. Another difference is that clients in Bay-County are granted an opportunity to pursue higher education compared with Central-County.

Clients praised Bay-County efforts toward granting them an opportunity to get an advanced degree, which in turn would enhance their prospects of landing a better job and would count toward their WTW work requirements. For example, an Asian single mother of a child, who works two part-time jobs and is pursuing her higher educational degree states,

30 hours [her work requirement] isn’t really fair for some people . . . because some people aren’t able to find a job or they can’t find childcare. I guess it varies. For me, I use both school and work, so I’m able to get it. Otherwise, I don’t think I could hit that 30 hours every single week. Especially if it’s part-time, their schedules always change.

## Discussion and Conclusion

While the existing literature on WTW employment is primarily focused on the demographic characteristics of clients (Achdut & Stier, 2016; Acs & Loprest, 2016; Hill, 2012), this study sheds light on the significance of structural place-based inequities and how that shapes both local-level WTW narratives on becoming job-ready and clients’ prospects of exiting WTW. The local WTW organizational narratives reproduce dominant macro-constructed policy discourses (Chang, 2019; Schneider & Ingram, 1993, 1997; Starke, 2020), namely, the notions of how “any job is better than no job” and clients’ “individual responsibility” for their own job prospects. This study is especially relevant now that the Covid-19 pandemic has plunged economies into recession. California, the study’s site, despite having one of the largest economies in the nation, displayed regional variation prior to the Covid-19 pandemic (Bohn et al., 2019). The study combines rich qualitative data both from an organizational and individual level and utilizes a comparative framework studying two varying economic contexts within California. The current study builds on the findings from the Chang et al. (2020) article by further discussing how county WTW narratives are contingent on local labor market conditions and how economic regional variation shapes the local agencies’ response and client experiences of becoming job-ready.

In the economically disadvantaged county, the organizational narrative of *getting and keeping any job*, *no matter how precarious*, was more dominant compared with the more economically privileged county. Based on the findings, we hypothesize, that this is largely due to the high unemployment rate in Central-County where there are fewer job opportunities to (re)integrate clients. Due to a dearth of livable wage job opportunities in this region, employers in this county context have an upper hand over clients as employees are ready to accept any working situation no matter how precarious or even abusive. Such a situation where employers are seen to have an advantage over their workers is resonant with other literature (Acs & Loprest, 2016). The hypothesis is further reiterated by clients who describe their subsidized and unsubsidized WTW employment opportunities as precarious, not adhering to standard schedules, and as being taking advantage of based on the client’s gender and race, in line with other qualitative research in this area (Cleaveland, 2007; Danziger et al., 2013; Seefeldt, 2017). Additional factors that could explain the dominance of this WTW narrative and the relative upper hand that employers appear to have in this region besides a high unemployment rate are the county’s more conservative politics, higher fiscal constraints on the local WTW-program, and clients being relatively lower skilled/educated (see Chang et al., 2020).

It is interesting to note that our findings reaffirm an earlier study (Lanfranconi et al., 2020b), in which we found that the more disadvantaged county operates on a more equity-oriented discourse, that is providing special attention to more disadvantaged clients, such as immigrant clients. Based on an analysis of Central-County administrative data, race also seems to play out less strongly in Central-County WTW practices (sanction and exemption) than it does in Bay-County (see Lanfranconi et al., 2020b). However, the dominance of the agency being embedded in a more disadvantaged economic context may shape the agency taking on a more punitive and stricter stance toward clients in terms of their WTW requirements. Such a strict stance places greater pressures on clients to accept unsafe and unhealthy work opportunities, while balancing familial stress and their own mental and physical well-being.

On the contrary, the economically privileged county largely adopts the narrative of *client’s individual responsibility*, that is, placing the onus of succeeding within WTW on the clients themselves much more so compared with the less economically privileged county. Hypothetically, this can be interpreted as the local agency responding to the economic context of a higher cost of living and soaring rent prices, where clients—despite holding multiple, challenging jobs—are unable to make ends meet and often experience homelessness or discrimination as a renter, as also found in Monroe and Tiller’s (2001) study about the numerous barriers that WTW clients face while seeking to exit WTW. Alternative explanations of the dominant WTW narrative may also be found in Bay-County’s urban location, near Silicon Valley where clients enter WTW with higher educational qualifications compared with Central-County clients; there is an expectation of clients being able to thrive in this context which enjoys comparatively lower unemployment and poverty rates. As per the individual responsibility narrative dominant within this region, clients are often made to reassess their lifestyle choices, adjust their standard of living, and are even encouraged to leave such an expensive region in pursuit of more stable housing. However, such a situation poses a paradox for families as moving to a less privileged economic context would imply fewer job opportunities as evidenced in the findings from Central-County.

Even though the more privileged county offers clients an opportunity for pursuing a higher educational degree, the county operates on a gender- and race-blind equality discourse, which treats all clients the same without recognizing the structural disadvantage that some clients face (see Lanfranconi et al., 2020b). Also, race seems to play out stronger in Bay-County’s WTW practices (sanction and exemption) than it does in Central County, as an analysis of Bay-County’s administrative data suggests (Lanfranconi et al., 2020b). As showcased in the current findings, clients have felt discriminated against in both their workplace as well as renters based on their race and ethnicity. Unstable housing impels WTW agencies to advice clients to move out of the county and to reconsider their life choices, meaning to lower their expectations of life choices, such as where to live or having another child, and improve their own human capital by upskilling/receiving education to become job-ready, as indicated in past research (Acs & Loprest, 2016; Cleaveland, 2007; Danziger et al., 2013).

The current study poses a few limitations and avenues for further research. The first limitation is that the sample size of clients spoken to was small: 14 clients out of two case-counties. However, both counties and clients were selected based on a purposive sampling as per clear criteria (Kelle & Kluge, 2010; TESOL International Association, 2021). The second limitation is the clients were not observed while working at their respective employment sites. Future studies could incorporate observations of WTW clients at their workplace to gain a more nuanced picture. The third limitation is the study did not include the employer’s perspective on hiring WTW clients, which could also be a future research topic to explore.

Overall, the findings emphasize the significance of how place-based inequities play out for clients, as their opportunities to become job-ready are very dependent on local employers, landlords, and local WTW agency narratives regarding WTW requirements. The article demonstrates how both local TANF organizational narratives and WTW client experiences are shaped to a considerable extent by the local economic context. This is especially true in a broader social-political context with poor employment protections especially for low-wage labor in the United States (Andersson et al., 2016; Loprest & Nichols, 2016). The findings also shed light on the fact that regardless of local agency setting, CalWORKs continues to operate in the TANF policy design social construction of how conditional welfare narratives continue to fall back on client individual responsibility and work-first (Chang, 2019; Chang & Romich, 2021; Schneider & Ingram, 1993, 1997; Starke, 2020). These discourses further shape the experiences of making clients job-ready and working largely in favor of low-wage employers and local landlords. Local organizational narratives and stringent WTW requirements impel clients to accept precarious, unsafe, and discriminatory low-wage working conditions or unstable housing conditions. Clients are taught to be grateful for being employed and often expected to lower their expectations around future job prospects and family planning. Variance of location and understanding structural barriers toward assisting clients become job-ready seems to be not taken into consideration, implying that conditional welfare falls back on its default limitations as highlighted in previous research (Grogger & Karoly, 2005; Pavetti, 2018; Starke, 2020).

### Implications for Practice

The findings provide various policy and practice implications. There is a need to review, revise, and readjust TANF benefits to the real cost of living, as higher benefits will help clients keep their jobs, especially in high-cost areas. While in Central-County the CalWORKs benefit level for a family of five is slightly higher than the median gross rent, the Bay-County CalWORKs benefit level is less than half of the median gross rent.

Given welfare devolution, local WTW agencies administering TANF need to operate flexibly instead of asking clients to be flexible. A forthcoming study (Lanfranconi & Basaran, forthcoming) suggests that frontline workers with social work educational background tend to be more flexible and client-driven than others, thereby providing greater impetus for additional intensive case management in the form of support services to TANF clients. In addition, agencies can improve their outreach to community colleges and vocational training centers and provide opportunities for clients to build their human capital as such opportunities would provide prospects of higher wage jobs.

As evidenced in the findings, agencies have tied up with employers who appear to have mistreated their WTW employees. Therefore, agencies need to conduct a thorough assessment of their local employer network to understand if they best serve clients’ interests and hold employers accountable, ensuring fair labor standards are being followed and prevent worker discrimination. In addition, agencies can seek out new employment avenues to obtain jobs that offer higher wages and better workplace standards.

Beyond TANF, there is a greater need for a higher minimum wage and more labor market control, as proposed by other OECD countries (OECD, 2020). Our study has shown it is difficult and often harmful to integrate clients in a labor market that is already overburdened. There is a need to consider alternative policy designs and constructions, which are based more on the idea of structural causes of poverty (Starke, 2020). Such alternative models have been proposed by Scandinavian welfare states, where besides a trend to more conditionality, the welfare state is still based on the principle of universalism and there are mostly no time limits in getting social assistance.

A more radical alternative to conditional welfare is universal basic income (UBI). UBI is a cash transfer given to all members of a community on a recurrent basis regardless of income level with no strings attached. Several UBI experiments that have been conducted across the globe showcase promising findings on how UBI seek to alleviate poverty, but UBI implementation needs to account for local contextual factors to maximize the benefits of a UBI (Hasdell, 2020).^4^ The social work profession is well poised to advocate for the uptake of unconditional welfare policy designs and conduct research to understand how such policy designs can assist people to become economically self-sufficient.

## Notes

1. Chang et al. (2020) identified in a cluster analysis of 58 Californian counties welfare-to-work (WTW) services four distinct WTW service delivery orientations: *employment-oriented*, *sanction-oriented*, *education-oriented*, and *training-oriented* service delivery systems.

2. See https://ca.db101.org/ca/programs/income_support/calworks/program2b.htm#:~:text=Besides%20cash%2C%20CalWORKs%20also%20provides,Health%20%26%20Disability%20Prevention%20(CHDP)

3. As is common in qualitative research, the sample size of most studies is limited (Mason, 2010). There are two important principles, however, to consider when doing qualitative research, to make the data meaningful: The first is purposive sampling, that is, to ensure diversity of opinions and experiences among study participants (Kelle & Kluge, 2010; Mason, 2010; TESOL International Association, 2021). The second is saturation, which means that collection of new data does not shed further light on the issue under investigation (Glaser & Strauss, 1967; Mason, 2010). Both these qualitative research principles have been applied to the current study.

4. Within California, the former mayor of the City of Stockton launched a guaranteed income initiative that had positive impacts on household income volatility, full-time employment, and financial scarcity among others shedding light on the promise of such initiatives (West et al., 2021). Currently, in the United States, there is a coalition of mayors called Mayors for a Guaranteed Income committed to launching guaranteed income pilots in their cities especially as a response to the pandemic’s economic downturn. See https://www.mayorsforagi.org/
